# Toughening Polylactic Acid with Ultrafine Fully Vulcanized Powdered Natural Rubber Graft-Copolymerized with Poly(styrene-co-acrylonitrile): Tailoring the Styrene–Acrylonitrile Ratio for Enhanced Interfacial Interactions

**DOI:** 10.3390/polym16162254

**Published:** 2024-08-08

**Authors:** Reza Gholami, Ibrahim Lawan, Sahar Ebrahimi, Achiraya Pattulee, Cheol-Hee Ahn, Sarawut Rimdusit

**Affiliations:** 1 Center of Excellence in Polymeric Materials for Medical Practice Devices, Department of Chemical Engineering, Faculty of Engineering, Chulalongkorn University, Bangkok 10330, Thailand; rezagholami215@gmail.com (R.G.); ilawan.age@buk.edu.ng (I.L.); ebrahimisahar2012@gmail.com (S.E.); achiraya.patt@gmail.com (A.P.); 2 Department of Materials Science and Engineering, Seoul National University, Seoul 08826, Republic of Korea; chahn@snu.ac.kr

**Keywords:** polylactic acid (PLA), bio-based toughening modifier, sustainable materials, ultrafine fully vulcanized powdered natural rubber, polymer composites

## Abstract

This study investigated the sustainable toughening of polylactic acid (PLA) by incorporating ultrafine fully vulcanized powdered natural rubber graft-copolymerized with poly-styrene-co-acrylonitrile (UFPNR-SAN). We investigated the effect of the styrene-to-acrylonitrile ratio (ST:AN) used during the grafting process on the final UFPNR-SAN compatibility with PLA. The ST:AN ratio was systematically varied during the grafting reaction to prepare UFPNR-SAN with a range of different surface energies. The ST:AN ratio of 4:1 showed the highest compatibility with the PLA matrix, attributed to optimal interfacial interactions and improved dispersion, as indicated by contact angle measurements and SEM observations. This resulted in a remarkable toughening of the PLA/UFPNR-SAN composite. For instance, an obvious fully ductile behavior without crack formation and flexural strain of around 17.5% against 5% of the neat PLA was recorded. In addition, 3.5 times improvement in the impact strength of the composite at 25 wt% dosage of the UFPNR-SAN was also achieved without compromising thermal properties. Overall, this study established the suitable ST:AN ratio on the grafting onto natural rubber to enhance interfacial interactions with PLA and its effects on the properties of the resulting PLA/UFPNR-SAN bio-based composite.

## 1. Introduction

Polylactic acid (PLA) has undergone extensive research over the past two decades due to its renewable and biodegradable properties [1,2,3,4,5], making it an ideal choice for eco-friendly product design to meet consumer demands and stringent regulations [6]. PLA’s sustainability, cost-effectiveness, and good mechanical properties make it a compelling alternative to conventional petroleum-based polymers [7,8]. This has gained significant attention from the automotive industry, particularly in producing car interior components [9,10]. Moreover, PLA is also a widely used thermoplastic in additive manufacturing processes (3D printing) [11]. Despite its advantages, PLA has limitations, including its inherent brittleness and poor impact strength. Several strategies have been employed to enhance its toughness including copolymerization [12,13], plasticization [14,15,16], the blending of PLA with various elastomers and polymers [17,18,19], and the incorporation of rigid fillers [20,21,22]. The most common and effective of these strategies is the blending of PLA and rubbers.

Natural rubber (NR), recognized as a sustainable bio-based elastomer, emerges as a promising solution for the sustainable toughening of PLA. However, the challenge arises from the inherent immiscibility of PLA and NR, leading to a coarse phase-separated morphology that reduces toughening efficiency [23]. To address this issue, ultrafine fully vulcanized powdered rubber (UFPR) offers a viable solution. UFPR, prepared through irradiation and spray drying processes, exhibits a unique core–shell elastomeric toughening filler with a significantly higher density of crosslinking on the particle surface [24]. Various commercial grades of UFPR have proven effective in modifying the toughness of different polymer matrices [25,26]. Zhao et al. [27] demonstrated the successful toughening of PLA using UFPR based on ethyl acrylate rubber, achieving remarkable enhanced tensile properties with just 1 wt% UFPR.

The transition to bio-based materials has motivated the exploration of bio-based UFPR fillers based on NR (UFPNR) [28,29,30,31,32], in which appreciable performance was reported for polybenzoxazine [32] and PVC [28]. The utilization of the UFPNR particles in PLA still presents the challenge of modifying UFPNR to tackle the issue of its compatibility in PLA. It is well established that interfacial interactions between particles and PLA significantly impact the filler–matrix compatibility and the particles’ dispersion quality [33,34]. Notably, these crucial interfacial interactions are directly linked to the surface energies of both PLA and the incorporated particles. Grafting approaches can be utilized to adjust the surface energy of UFPNR to improve compatibility with PLA. Longsiri et al. [29] grafted poly-styrene-co-acrylonitrile (PSAN) onto NR to produce UFPNR graft-copolymerized with PSAN (UFPNR-SAN), resulting a core–shell structure with NR as the core and PSAN as the shell, and, therein, it was established that varying the styrene-to-acrylonitrile (ST:AN) ratio resulted in a variation in the water contact angle of the UFPNR-SAN. The findings suggest that by changing the ST:AN ratio during the grafting process, the surface energy and polarity of the final UFPNR-SAN can be controlled. This is because AN is highly polar and ST is a nonpolar monomer. This variation in polarity offers a pathway to tailor the interfacial interactions between UFPNR-SAN and PLA.

Various approaches have been explored to enhance PLA’s toughness. However, the development of sustainable toughening strategies remains an active area of research. One promising approach could be incorporating UFPNR-SAN into PLA. However, to the best of our knowledge, no previous studies have explored incorporating UFPNR-SAN into PLA.

Therefore, the main objective of this research was to investigate the use of UFPNR-SAN as a bio-based toughening agent for PLA. Through this objective, we aimed to determine the optimal ST:AN ratio and weight content for maximizing PLA toughness while minimizing negative impacts on other physical properties. Our research consisted of two main steps. Firstly, UFPNR-SANs with varying ST:AN ratios were prepared and characterized by evaluating their surface energy and interfacial interactions with PLA. Secondly, PLA/UFPNR-SAN composites were prepared by mixing each UFPNR-SAN formulation with PLA at different weight percentages. PLA/UFPNR-SAN composites were characterized using morphological analysis, mechanical tests, thermal analysis, and dynamic mechanical analysis.

## 2. Materials and Methods

### 2.1. Materials Used

PLA 4043D was obtained from NatureWorks, Plymouth, MN, USA. Deproteinized natural rubber (DPNR) SELATEX 3821 was supplied by Unimac Rubber Co., Ltd., Na To Ming, Thailand. Sodium dodecyl sulfate (SDS; 99%, Merck, Darmstadt, Germany), ST (99%), AN (99%), tert-butyl hydroperoxide (TBHPO; 70% in water), tetraethylenepentamine (TEPA; 94% in water), di-trimethylolpropane tetra-acrylate (DTMPTA), glycerol, and di-iodomethane were purchased from Tokyo Chemical Industry Co., Ltd. (Tokyo, Japan).

### 2.2. Preparation of Ultrafine Fully Vulcanized Powdered Natural Rubber Grafted-Copolymerized with Poly(styrene-co-acrylonitrile) (UFPNR-SAN)

**Figure 1:**
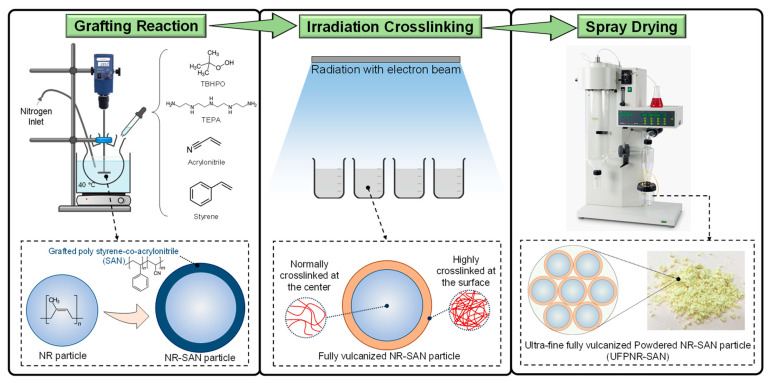
Schematic illustration of UFPNR-SAN production process.

UFPNR-SAN was prepared based on previous work carried out by Longsiri et al. [29]. Accordingly, the schematic of production process is illustrated in Figure 1. The graft-copolymerization procedure involving the incorporation of ST and AN onto DPNR took place in a latex state. The reaction was carried out within a 500 cm^3^ glass reactor equipped with a mechanical stirrer, water bath, and a nitrogen gas inlet. The DPNR latex and SDS were added into the reactor under a nitrogen atmosphere and were stirred at 400 rpm and 40 °C for 2 h. Initiators, namely TEPA and TBHPO, were utilized at a concentration of 3.5 × 10^−5^ mol/g of dry rubber content (DRC). ST and AN monomers were incorporated into the solution in varying weight ratios (namely, 1:0, 4:1, 1:1, 1:4, and 0:1) at a total concentration of 0.5 × 10^−3^ mol/g of dry rubber (equivalent to 5 phr). The grafting reaction proceeded for an additional 2.5 h.

Following the grafting reaction, the grafted NR underwent a dilution to reach 20% DRC using deionized water, and 3 phr of DTMPTA was added as a coagent. The latex mixture was stirred for 15 min prior to vulcanization through electron beam irradiation at a dose of 300 kGy. Subsequently, the vulcanized grafted NR was subjected to a drying process using a spray dryer to achieve the desired product, UFPNR-SAN. The specific spray dryer utilized was the B-290 model from BUCHI (Flawil, Switzerland). The process involved setting the inlet temperature at 150 °C, maintaining a feed flow rate of 7 mL/min, and an air flow rate of 500 L/h.

### 2.3. Preparation of PLA/UFPNR-SAN Composites

The mixing process was conducted using an internal mixer from Chareon TUT Co., Ltd., Samutprakarn, Thailand, at a temperature of 170 °C and a rotor speed of 80 rpm. This mixing was carried out for 10 min. To ensure the elimination of any residual moisture, both the PLA pellets and the UFPNR-SAN powders were pre-dried at 80 °C for 24 h before the mixing process. After the mixing step, the flexural and Izod specimens were molded separately utilizing the compression molding technique, according to the ASTM D4703 standard [35]. Before the molding process, the PLA/UFPNR-SAN (denoted as PSANx:x, where x:x represents the weight ratio of ST:AN) compounds were dried at 80 °C for 24 h. The compression molding consisted of a two-step procedure: first, pressing between hot plates at 170 °C for 2 min under a pressure of 100 bar, and subsequently, cold pressing to rapidly reduce to room temperature within two minutes, while maintaining a pressure of 100 bars. An identical methodology was employed to produce neat PLA specimens for the purpose of performing a comparative analysis. To ensure that void content did not significantly influence the mechanical properties (target < 5%), we measured the void content in PLA/UFPNR-SAN composites across different weight fractions. The results showed a negligible void content (~1%). The detailed experimental procedures and data are provided in the Supplementary Materials.

### 2.4. Characterizations

#### 2.4.1. Estimation of Interfacial Interactions between PLA and UFPNR-SAN Using Contact Angle Measurements

Contact angle measurements were carried out using the sessile drop technique on a contact angle meter (KYOWA DMe-210, Osaka, Japan), following the standard ISO 19403-2 guidelines [36]. Before taking the measurements, rubber films for each UFPNR-SAN were created through the solvent casting of latex, while PLA films were produced using compression molding. For the contact angle measurements, three different probe liquids were utilized with known dispersion (*σ^d^*) and polar (*σ^p^*) components of surface tension: water (*σ^d^* = 21.8 and *σ^p^* = 51 mN/m), di-iodomethane (*σ^d^* = 52.8 and *σ^p^* =0 mN/m), and glycerol (*σ^d^* = 37 and *σ^p^* = 26.4 mN/m). A total of five separate contact angle measurements were conducted for each test liquid on each sample, and the average value was employed to determine the surface energy. This was achieved by applying Equation (1) to each liquid and performing a linear regression to obtain the desired parameters [36].

(1) $$\left(1+\cos ϴ\right)\sigma_{L}=2\left[{\left(\sigma_{s}^{d}\sigma_{L}^{d}\right)}^{0.5}+{\left(\sigma_{s}^{p}\sigma_{L}^{p}\right)}^{0.5}\right]$$

where *ϴ* represents the contact angle between the liquid and the polymer film; $\sigma_{L}$ and $\sigma_{s}$ are the surface energies of the liquid and solid, respectively; and the d and p superscripts denote the dispersive and polar components of the surface energy, respectively. While the contact angle measurements were conducted at room temperature (25 °C), extrapolating the obtained surface energies to the processing temperature is necessary. This involved assuming constant thermal coefficients for surface energy $\left(\frac{d\sigma }{dT}\right)$ and polarity ratio $\left(\frac{\sigma^{p}}{\sigma }\right)$. In this study, the reported $\frac{d\sigma }{dT}$ value for PLA was used [33]. However, for PSAN (the shell of UFPNR-SAN), a simple rule of mixture based on values reported for polystyrene and polyacrylonitrile was employed [37]. Finally, the interfacial tension γ and work of adhesion W between two components were estimated using Equations (2) and (3), respectively [33].

(2) $$\gamma_{A-B}=\sigma_{A}+\sigma_{B}-2\left[{\left(\sigma_{A}^{d}\sigma_{B}^{d}\right)}^{0.5}+{\left(\sigma_{A}^{p}\sigma_{B}^{p}\right)}^{0.5}\right]$$

(3) $$W_{A-B}=2\left[{\left(\sigma_{A}^{d}\sigma_{B}^{d}\right)}^{0.5}+{\left(\sigma_{A}^{p}\sigma_{B}^{p}\right)}^{0.5}\right]$$

where σ is surface energy, and the d and p superscripts denote the dispersive and polar components of the surface energy, respectively. The *A* and *B* subscripts represent PLA and UFPNR-SAN particles, respectively. Equation (3) can also be used to estimate the work of cohesion between the UFPNR-SAN particles ($W_{B-B}$), which simply equals ${2\sigma }_{B}$. In order to study the interplay between the work of adhesion and the work of cohesion, the dispersion factor was calculated using Equation (4) [33].

(4) $$Dispersion\;factor=\frac{work\;of\;adhesion\;(W_{A-B})}{work\;of\;cohesion\;(W_{B-B})}$$

The dispersion factor effectively serves as a measure of the adequacy of interfacial adhesion in transferring shear forces to UFPNR-SAN aggregates during melt blending and facilitating their separation into individual particles. Essentially, values exceeding 1 provide assurance that the interfacial adhesion is sufficient to overcome the internal cohesive forces within the particle aggregates, promoting effective dispersion.

#### 2.4.2. Morphological Characterization of the Composites

Scanning electron microscopy (SEM) was employed to examine the morphological microstructure of UFPNR-SAN particles within the PLA composites. For this purpose, specimens were kept in liquid nitrogen for 30 min and then fractured cryogenically. Prior to SEM analysis, a thin layer of gold was sputtered onto the cryofractured surfaces and then examined at an accelerated voltage of 10 kV. The same sample preparation procedure was replicated for the Izod fracture surfaces to investigate the morphology of the fracture surface in response to impact failure.

#### 2.4.3. Mechanical Tests of the Composites

The flexural properties of the PLA/UFPNR-SAN samples were evaluated through three-point bending tests using an INSTRON 5567 (Norwood, MA, USA) universal testing machine. The flexural test was conducted according to the ASTM D790 standard [38], and flexural specimens with a size of 90 × 12.7 × 3.2 mm^3^ were produced via compression molding. The displacement speed was set at 15 mm/min and the span length at 52 mm. The test proceeded until a load loss of 50% to observe the post-yield performance of each specimen. However, only the data from the initial part of the test were used for the calculation of flexural properties. Moreover, a notched Izod impact test was performed following the guidelines outlined in ASTM D256 [39] using a pendulum impact tester (Tinius Olsen IT503/504, Royal Oak, MI, USA). To ensure the accuracy and reliability of the results, each test was repeated five times for the flexural and Izod impact tests, and the outcomes were averaged.

#### 2.4.4. Thermal Analysis of the Composites

Differential scanning calorimetry (DSC) analysis was performed using a DSC1 module from Mettler-Toledo, Greifensee, Switzerland, to study the thermal properties and crystallinity of the PLA/UFPNR-SAN composites. Molded specimens weighing 5 mg were subjected to a heating cycle from 30 to 190 °C at a rate of 10 °C/min under a nitrogen atmosphere. Data from the first heating cycle were recorded to determine the degree of crystallinity, which can be calculated using Equation (5):

(5) $$Degree\;of\;crystalinity\left(\%\right)=\left(\frac{{\Delta H}_{m}-{\Delta H}_{c}}{w_{f}{\Delta H}_{m}^{100\%}}\right)\times 100$$

where ${\Delta H}_{m}$ and ${\Delta H}_{c}$ represent the enthalpy of melting and cold crystallization, respectively, during first heating cycle, which can be obtained by calculating the area under each peak. ${\Delta H}_{m}^{100\%}$ is the enthalpy of 100% crystalline PLA homopolymer, which is equal to 93.7 J/g [40,41], and $w_{f}$ represents the weight fraction of the PLA component in the composite.

Furthermore, the thermal stability of the composites was investigated using a thermogravimetric analyzer (model TGA1 module from Mettler-Toledo, Greifensee, Switzerland). Samples weighing approximately 10 mg were subjected to a heating cycle from 30 °C to 650 °C at a rate of 20 °C/min under a nitrogen atmosphere.

#### 2.4.5. Dynamic Mechanical Analysis of the Composites

DMA was conducted using a DMA1 module from Mettler-Toledo, Switzerland, to study the viscoelastic properties of the PLA/UFPNR-SAN composites. A dual cantilever beam clamp was used for the DMA test, and samples (dimensions 50 × 10 × 1.5 mm^3^) were tested under a load-control mode at the frequency of 1 Hz and oscillatory displacement with an amplitude of 15 µm at a 2 °C/min heating rate from 30 to 100 °C.

## 3. Results and Discussion

### 3.1. Interfacial Interactions between PLA and UFPNR-SAN

**Figure 2:**
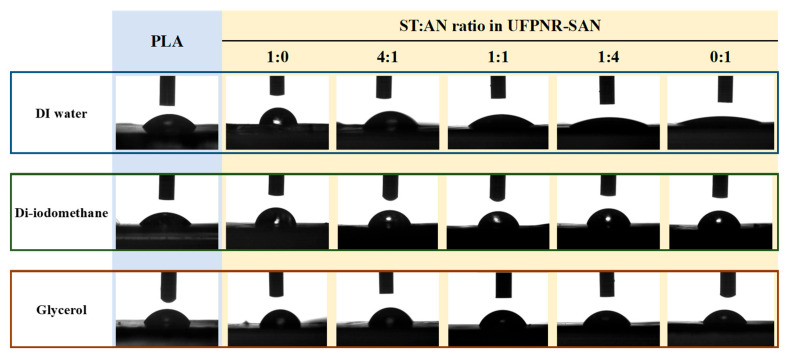
Typical measured contact angles of different test liquids on PLA (blue colored frame) and UFPNR-SAN samples at varying ratios of ST:AN (yellow colored frame).

**Figure 3:**
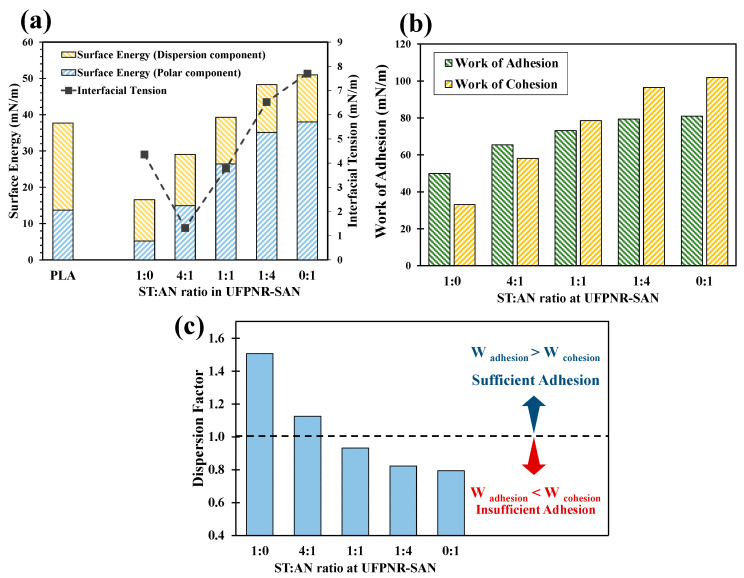
Interfacial interaction parameters in PLA/UFPNR-SAN composites at 170 °C: (**a**) surface energies of PLA and UFPNR-SAN and interfacial tension between them, (**b**) work of adhesion at PLA/UFPNR-SAN interface and interparticle work of cohesion, (**c**) dispersion factor of UFPNR-SAN at different ST:AN ratios.

The typical contact angle images recorded for PLA and each UFPNR-SAN formulation with DI water, di-iodomethane, and glycerol are presented in Figure 2. The contact angle values measured and the interfacial interaction parameters calculated are summarized in Table 1 and Table 2, respectively. The significant variation in DI water and glycerol contact angles due to the different ST:AN ratios used in grafting onto NR resulted in remarkable changes in the surface energy and interfacial tension, as it can be seen in Figure 3a. The results showed an increasing trend in the polar component of the surface energy with increasing AN content, attributed to the fact that AN is a highly polar monomer compared to nonpolar ST. Notably, the 4:1 ST:AN ratio exhibited the closest polarity and surface energy to PLA, leading to the minimum interfacial tension between UFPNR-SAN and PLA. This suggests enhanced compatibility; however, low interfacial tension alone is not sufficient for optimal dispersion of particles within PLA [33]. Therefore, further investigation of the interfacial adhesion between UFPNR-SAN particles and PLA is necessary.

Figure 3b depicts the work of adhesion and cohesion values for different UFPNR-SAN formulations. At the processing temperature, the work of adhesion between PLA and UFPNR-SAN particles showed a slight increase with increasing AN content. Conversely, the work of cohesion within the UFPNR-SAN particles exhibited a considerably larger increase with increasing AN content, surpassing the work of adhesion for ST:AN ratios exceeding 1:1. This trend becomes more evident in Figure 3c, in which the dispersion factor (adhesion divided by cohesion) only surpasses 1 for the 1:0 and 4:1 formulation. While the 1:0 UFPNR-SAN formulation showed the highest dispersion factor, its low interfacial adhesion might compromise mechanical properties at application, making it potentially less desirable. Conversely, the 4:1 ratio emerged as the most favorable formulation of UFPNR-SAN due to its dispersion factor exceeding 1, guaranteeing sufficient interfacial adhesion with PLA for overcoming cohesive forces within the UFPNR-SAN particles during melt blending, while maintaining adhesion values comparable to the maximum observed.

Overall, the findings demonstrated that the surface energy of the final UFPNR-SAN product was effectively manipulated by adjusting the ratio of two monomers, ST (nonpolar) and AN (polar), during the grafting reaction onto NR. By considering these variations in the surface energies, the interfacial interaction parameters could be optimized. Therefore, the 4:1 ST:AN formulation held the most promise for achieving optimal compatibility and interfacial interactions between UFPNR-SAN and PLA. This formulation, along with its counterpart (1:4 ST:AN ratio), was selected for mixing with PLA at different weight contents for further comparative studies.

### 3.2. Morphological Analysis of the Composites

**Figure 4:**
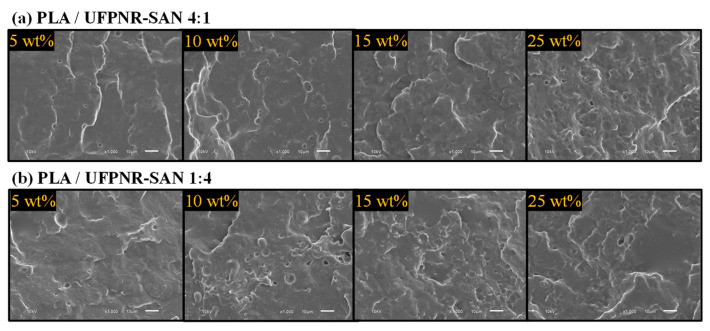
SEM micrographs of cryofractured surface of PLA blends with different weight contents of UFPNR-SAN with ST:AN ratios of (**a**) 4:1 and (**b**) 1:4.

The SEM images of the cryofractured surfaces of the PLA/UFPNR-SAN composites with two formulations of 4:1 and 1:4 ratios of ST:AN are depicted in Figure 4. Upon careful examination, it became evident that UFPNR-SAN particles with a 4:1 ratio exhibited a better dispersion quality. In this case, as it can be seen in Figure 4a, individual particles, characterized by their spherical shape, were distinctly identifiable across all weight percentages. However, when the ST:AN ratio was adjusted to 1:4, the dispersion of the UFPNR-SAN particles was less uniform, particularly at the 15% and 25% weight contents. Here, distinguishing individual particles became more challenging, while clusters of particles and areas devoid of distributed particles became more apparent. This observation suggests a nonuniform dispersion pattern.

The shift in dispersion behavior observed when the ST:AN ratio transitioned from 4:1 to 1:4 stemmed from differences in interfacial interactions between the particles and PLA, as discussed in the previous section. Analysis of the interfacial interaction parameters suggested that the UFPNR-SAN 4:1 formulation should exhibit better dispersion. This expectation was based on its minimal interfacial tension and, crucially, its sufficient interfacial adhesion for effectively transferring shear forces to particle agglomerates during melt blending. This translated to a dispersion factor of 1.16, compared to the 0.82 value for UFPNR-SAN 1:4. These findings align with previous observations in PLA composites filled with nanocrystalline cellulose (NCC), in which NCC particles with dispersion factors exceeding 1 demonstrated superior dispersion compared to those with values below 1 [33].

### 3.3. Mechanical Properties of the Composites

**Figure 5:**
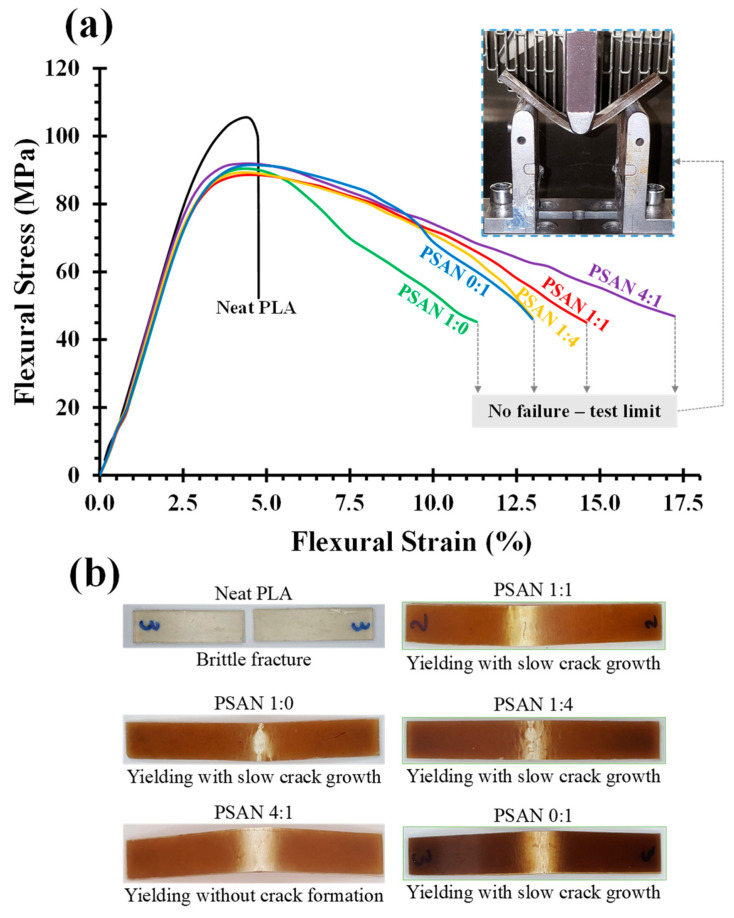
(**a**) Flexural stress–strain curves of PLA/UFPNR-SAN blends at 5 wt% with varying ST:AN ratios. (**b**) Deformed regions on the back face of the flexural specimens.

The results of the flexural and Izod impact tests for PLA/UFPNR-SAN composites with different ST:AN ratios and weight contents are summarized in Table 3. The flexural modulus and strength of PLA/UFPNR-SAN at a fixed 5 wt% of particles resulted in a marginal decrease of 5% and 15%, respectively, for all ST:AN ratios. The remarkable similarity in flexural modulus and strength among the different ST:AN ratios can be attributed to the fact that these properties are primarily influenced by material behavior before yielding. Consequently, it was expected that these properties would exhibit relative consistency across the various ST:AN ratios within the UFPNR-SAN composite, despite the identical weight content. However, the post-yield performance of the blends exhibited different behaviors. Figure 5a clearly represents the typical flexural stress–strain curves, highlighting the differences in failure behaviors among the specimens. Neat PLA specimens, characterized by their inherent brittleness, displayed sudden crack propagation and fracture. In contrast, UFPNR-toughened PLA specimens did not undergo fracture until the test limit (i.e., 50% of maximum load) and showed considerable ductile behavior, as shown in the inset image of a typical fully deformed sample in Figure 5a. However, this ductile behavior exhibited different post-yield performance in the stress–strain curves due to different modes of failure. Figure 5b shows the deformed region of the back face of each specimen. Among all specimens, PLA/UFPNR with a 4:1 ST:AN ratio (PSAN4:1) exhibited fully ductile behavior such that it underwent yielding without the formation of any considerable crack, resulting in a slightly larger whitening region. In contrast, other specimens exhibited yielding with the formation and slow growth of cracks. This difference in the mode of failure was reflected in the stress–strain curve, where the drop in load was faster in specimens with the formation and growth of cracks than in the PSAN4:1 specimen, which did not show any crack formation.

**Figure 6:**
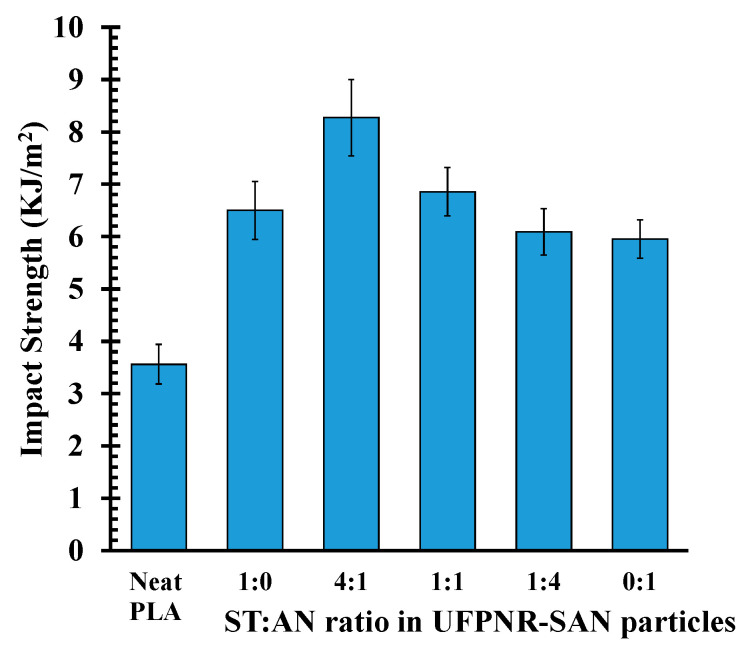
Impact strength of PLA blends with UFPNR-SAN at varying ST:AN ratios and fixed 5 wt%.

The effect of the ST:AN ratio on the toughness improvement of PLA/UFPNR-SAN composites was more pronounced in the Izod impact strength results. Figure 6 shows the impact strength of PLA blends with UFPNR-SAN at varying ST:AN ratios. The incorporation of just 5 wt% UFPNR-SAN particles resulted in an enhancement in impact strength. Notably, the UFPNR-SAN with 4:1 ST:AN ratio exhibited the most improvement among all the ratios employed for grafting onto natural rubber, increasing the impact strength by 2.3 times that of neat PLA. This improvement compares favorably to the reported improvement achieved with commercialized UFPR based on synthetic acrylate rubber at the same 5% UFPR dosage [27].

**Figure 7:**
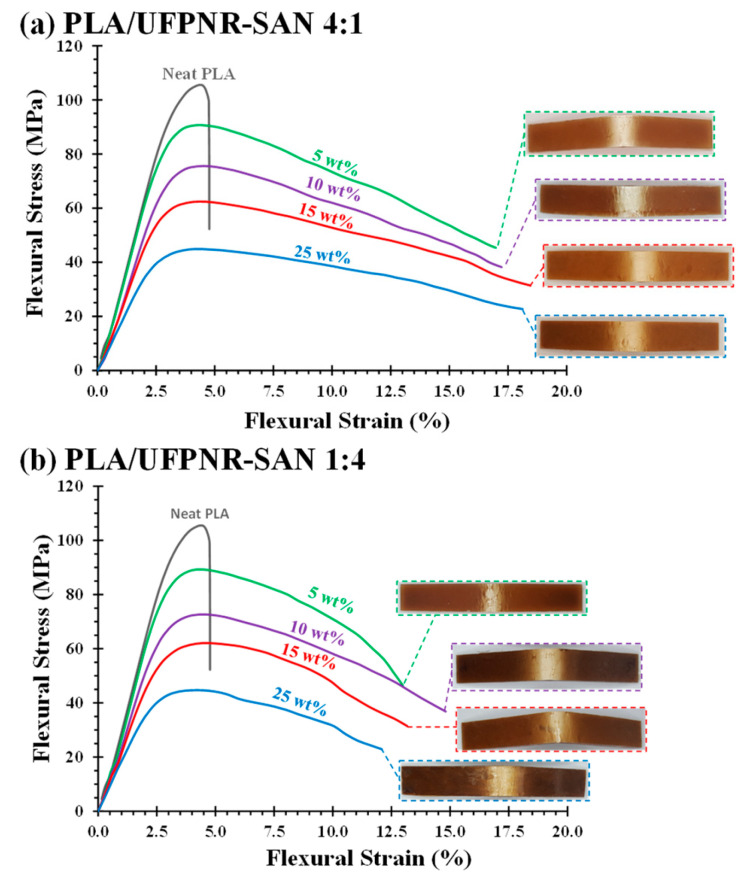
Flexural stress–strain curves of PLA/UFPNR-SAN blends with varying weight contents at ST:AN ratios of (**a**) 4:1 and (**b**) 1:4.

To investigate the effect of the ST:AN ratio in PLA/UFPNR-SAN at higher weight contents exceeding 5 wt%, UFPNR-SAN particles with a 4:1 ST:AN ratio were selected, as this is considered the optimal ratio for enhancing compatibility with PLA. Additionally, the 1:4 ST:AN ratio counterpart was included for comparative analysis. Figure 7 shows the typical stress–strain curves obtained from flexural tests of PLA/UFPNR-SAN composites with ST:AN ratios of 4:1 and 1:4 at different weight contents. As shown in Table 3 and Figure 7, the flexural strength and modulus decreased with the addition of more UFPNR-SAN particles, irrespective of the particle type. Generally, an improvement in toughness through the addition of rubbery modifiers often correlates with reductions in stiffness and hardness, which is a typical trade-off when enhancing toughness in polymer composites. This aligns with observations reported in the literature [27,42,43]. As previously observed and explained, there was no significant difference in flexural strength and modulus between the two types of UFPNR-SAN (i.e., 4:1 or 1:4 ST:AN ratio). However, each UFPNR-SAN type exhibited remarkably different failure behavior in the flexural and Izod impact tests. The PLA/UFPNR-SAN 4:1 specimen showed fully ductile behavior at all weight contents (Figure 7a), with no crack formation after yielding in the deformation zone on the back face of specimens until the test limit. In contrast, the PLA/UFPNR-SAN 1:4 blends (Figure 7b) showed yielding accompanied by crack formation and crack growth, leading to a faster reduction in load and reaching the test limit at smaller strains compared to the PLA/UFPNR-SAN 4:1 composite.

**Figure 8:**
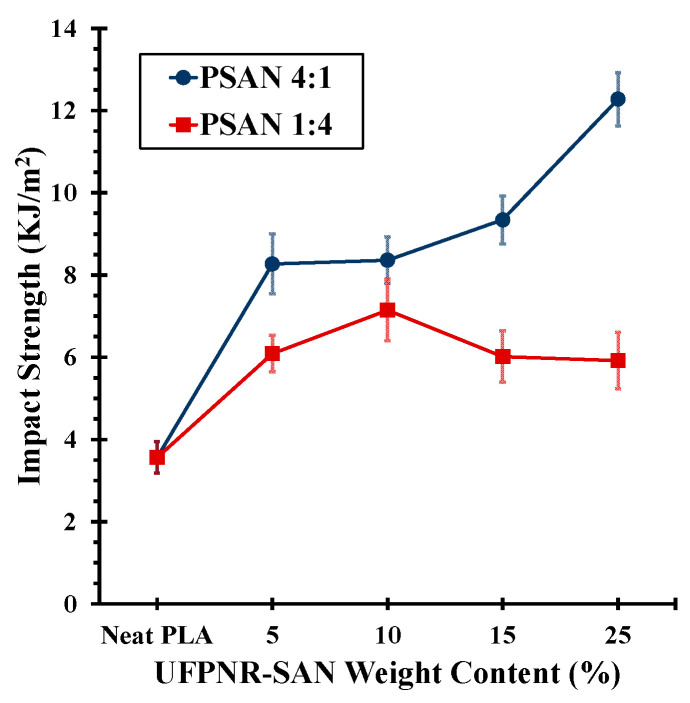
Impact strength of PLA blends with UFPNR-SAN with ST:AN ratios of 4:1 and 1:4 at varying weight contents of UFPNR-SAN.

The superiority of the 4:1 ST:AN ratio in UFPNR-SAN over other ST:AN ratios is clearer in the Izod impact test results (Figure 8). For the PSAN1:4 samples, the maximum improvement in impact strength was 101% at 10 wt%, but it began to decrease beyond this weight content. In contrast, for the PSAN4:1 specimen, the trend showed a consistent increase, with a maximum improvement of 245% at 25 wt% of UFPNR-SAN with an ST:AN ratio of 4:1.

**Figure 9:**
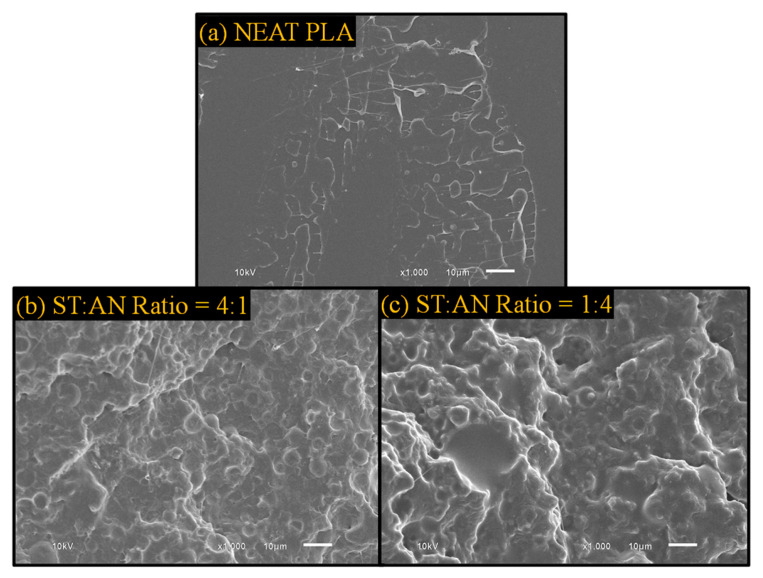
SEM micrographs of Izod fracture surface of (**a**) neat PLA and PLA/UFPNR-SAN composites with ST:AN ratios of (**b**) 4:1 and (**c**) 1:4 at 25 wt%.

The superior performance of the UFPNR-SAN 4:1 formulation in comparison to that of the UFPNR-SAN 1:4 can be explained by referring to Figure 9. This figure presents SEM micrographs illustrating the Izod fracture surfaces of neat PLA as well as PLA blends containing 25 wt% of UFPNR-SAN with varying styrene and acrylonitrile (ST:AN) ratios of 4:1 and 1:4. As anticipated, neat PLA displayed a smooth fracture surface due to its inherent brittleness. However, when UFPNR-SAN particles were incorporated, both blends exhibited rougher fracture surfaces, indicating improved toughness. Notably, the dispersion of UFPNR-SAN particles mirrored what we observed in the SEM analysis of cryofracture surfaces in Section 3.2. Specifically, UFPNR-SAN with an ST:AN ratio of 4:1 displayed a uniformly dispersed pattern of individual particles. This uniform dispersion of particles helped to achieve a uniform load transfer from the PLA matrix to the UFPNR-SAN particles leading to a higher energy dissipation and consequently a higher toughness. In contrast, UFPNR-SAN with an ST:AN ratio of 1:4 exhibited nonuniform dispersion, with noticeable agglomerations of rubber particles. This was attributed to the better compatibility of the UFPNR-SAN 4:1 formulation with PLA compared to others.

### 3.4. Crystallization Analysis and Thermal Properties of the Composites

**Figure 10:**
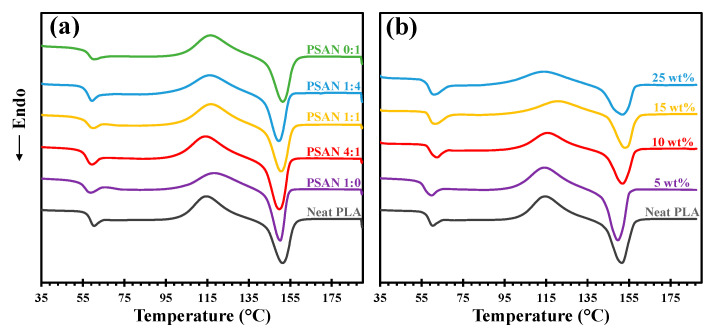
DSC thermograms of PLA/UFPNR-SAN blends: (**a**) at fixed 5 wt% of UFPNR-SAN with various ST:AN ratios and (**b**) with a fixed 4:1 ST:AN ratio and different weight contents.

Figure 10a presents the DSC curves recorded from PLA/UFPNR-SAN composites with varied ST:AN ratios at a fixed weight content of 5 wt%. It is evident that the addition of 5 wt% of UFPNR-SAN did not induce any significant alterations in the thermographs or the glass transition temperature (T_g_) and melting peak temperature (T_m_) values compared to the neat PLA. Each sample exhibited an exothermic peak associated with cold crystallization, followed by an endothermic melting peak. The areas under these peaks, representing the enthalpy of cold crystallization (Δ*H_c_*) and melting (Δ*H_m_*) as detailed in Table 4, were remarkably similar. These results indicated that the PLA phase in the molded samples remained in an amorphous state, with no crystalline phase formation. This behavior can be attributed to the high cooling rate employed during the molding process.

Furthermore, as shown in Figure 10b, the thermal transition properties of the PLA phase with varying weight contents of UFPNR-SAN exhibited similar trends, with T_g_ and T_m_ remaining comparable to that of neat PLA. However, PLA cold crystallization was hindered by an increase in the UFPNR-SAN weight content, as evidenced by the diminishing intensity of the cold crystallization peak due to the dilution effect of the PLA phase. Despite this observed variation in the cold crystallization peak, the area under both the cold crystallization peak and the melting peak remained constant across all weight contents, implying the absence of significant crystalline phase formation.

Observations from the DSC thermograms indicated that the degree of crystallinity in the PLA phase of all the molded specimens was not significantly influenced by either the ST:AN ratio in UFPNR-SAN or the weight content. Therefore, it can be concluded that the enhanced toughness and ductility observed in PLA/UFPNR-SAN blends were exclusively attributed to the toughening mechanisms associated with UFPNR-SAN incorporation into PLA, as well as the favorable microstructure of the composites arising from optimized interfacial interactions during melt blending.

**Figure 11:**
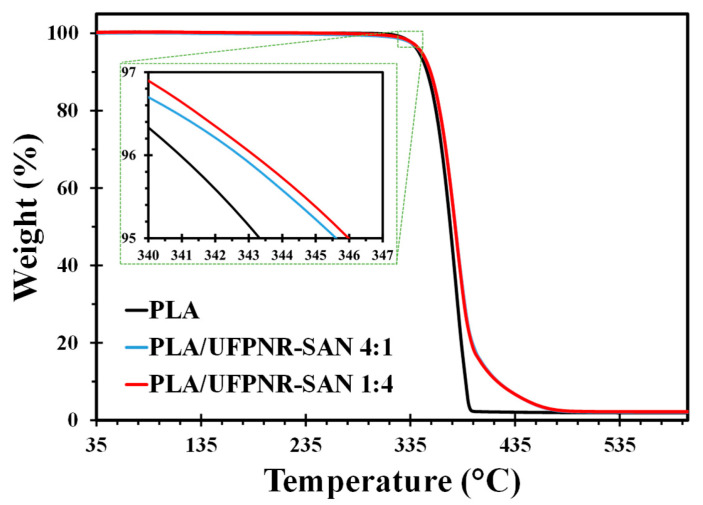
TGA thermograms of PLA/UFPNR-SAN blends at fixed 25 wt%.

The thermal decomposition behavior of neat PLA and PLA/UFPNR-SAN blends at the highest weight content of each UFPNR-SAN type (i.e., 25 wt%) was investigated using thermogravimetric analysis (TGA). As shown in Figure 11, the temperature at which 5% weight loss occurred (T_d5_) for neat PLA was 343 °C, while the Td values for the PLA/UFPNR-SAN blends were 345 °C at a 4:1 ST:AN ratio and 346 °C at a 1:4 ST:AN ratio. This indicates that the addition of UFPNR-SAN marginally increased the thermal stability of PLA.

The results of the TGA and DSC analyses suggested that, while UFPNR-SAN did not significantly improve the thermal properties of PLA, it did not have any adverse impact on other physical properties. This is consistent with the goal of improving toughness while minimizing losses in other desirable properties.

### 3.5. Viscoelastic Properties of the Composites

**Figure 12:**
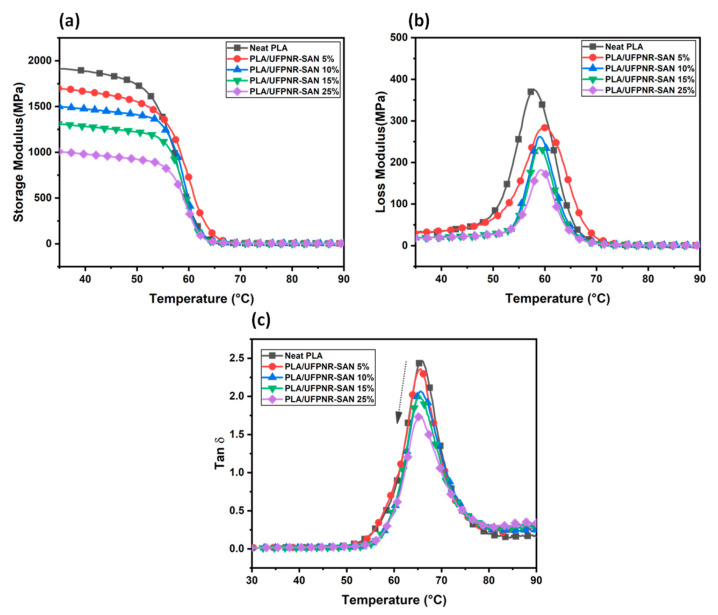
DMA curves of PLA and PLA/UFPNR-SAN 4:1 composite with varying wt% of UFPNR-SAN: (**a**) Storage modulus. (**b**) Loss Modulus. (**c**) Loss factor (arrow shows the decreasing trend of the peak height by increasing UFPNR-SAN weight content).

DMA analysis was employed to determine the storage modulus (E′), loss modulus (E′′), and damping parameter (tan δ = E′′/E′) values for PLA/UFPNR-SAN 4:1 (as the optimum formulation of UFPRN-SAN), as illustrated in Figure 12. This analysis was performed to investigate the influence of the UFPNR-SAN toughening agent on the viscoelastic properties of PLA.

The material’s elasticity behavior is typically indicated by the E′. The E′ profiles of all blends (Figure 12a) exhibited a consistent shape, featuring a mild decrease with increasing temperature coming from the softened chain segments from initial temperature to 50 °C, followed by a pronounced sharp decrease in the temperature range of 50−65 °C attributable to glass transition phenomena. The initial E′ value for pure PLA stood at 1.95 GPa, which was reduced to a range of 1 GPa by the consistent incorporation of UFPNR-SAN. This is a normal phenomenon in rubber-toughened systems, and this reduction in the storage modulus corresponded to what was observed for the flexural modulus.

E″ indicates the material’s ability to dissipate energy as heat, while tan δ represents the ratio of this dissipated energy relative to the energy stored during the loading cycle. The peak values of both E″ and tan δ correspond to the Tg of the material in different perspectives. As shown in Figure 12b,c, the E″ peak for PLA and the PLA/UFPNR-SAN composites remained relatively constant around 58–60 °C, exhibiting no significant change with increasing UFPNR-SAN content. Similarly, the tan δ peak consistently occurred at approximately 65 °C, regardless of the composition of the PLA composites. However, the peak heights of both E′′ and tan δ decreased as the weight content of UFPNR-SAN increased. This behavior has been reported in PLA toughened with rubbery agents [27,42,43] and can be attributed to the hindered chain mobility and reduced mechanical loss associated with overcoming intermolecular chain frictions [40]. While the T_g_ values obtained from E″ and tan δ differed from each other and from those obtained from DSC, this discrepancy can be ascribed to the underlying principles of each method. Notably, the independence of PLA’s T_g_ from the incorporation of UFPNR-SAN particles aligns with the T_g_ values obtained from DSC.

## 4. Conclusions

Based on the results recorded and discussion made regarding the sustainable toughening for polylactic acid (PLA) by the incorporation of UFPNR-SAN particles, the following conclusions could be drawn:

1. Adjusting the ST:AN ratio during the grafting reaction onto NR effectively tailored the surface energy of the UFPNR-SAN particles, influencing their interfacial interactions with PLA. UFPNR-SAN particles with a 4:1 ST:AN ratio exhibited optimal compatibility with PLA, due to their minimum interfacial tension with PLA as well as their sufficient particle–matrix interfacial.
2. SEM analysis revealed that UFPNR-SAN particles with a 4:1 ST:AN adhesion at processing temperature ratio exhibited comparatively superior dispersion within the PLA matrix. This enhanced dispersion was attributed to the aforementioned enhanced compatibility.
3. The significance of the ST:AN ratio in UFPNR-SAN formulations became evident in flexural tests. Specifically, the 4:1 ST:AN ratio demonstrated optimal interfacial interactions, leading to fully ductile behavior in flexural tests. This formulation exhibited superior performance by preventing crack formation at the back face of the flexural specimens, distinguishing it from other ratios.
4. The superiority of the 4:1 ST:AN ratio extended to the Izod impact strength, with UFPNR-SAN at this ratio indicating the highest improvement in impact strength at a fixed weight content of 5 wt%. This superiority persisted in PLA/UFPNR-SAN composites with higher weight contents of UFPNR-SAN.
5. Adjusting UFPNR-SAN particles by grafting the appropriate ratio of ST and AN monomers with different polarities proved to be a successful strategy for optimizing interfacial interactions, leading to higher level of compatibility, improved morphology, and ultimately higher toughness at identical weight contents of toughening fillers.
6. The DSC thermograms revealed that the degree of crystallinity in the PLA phase of the molded specimens was not notably affected by the ST:AN ratio in UFPNR-SAN or by its weight content. Consequently, the improved toughness and ductility observed in PLA/UFPNR-SAN composites were exclusively attributed to the toughening mechanisms of UFPNR-SAN incorporated into PLA, along with the favorable microstructure resulting from optimized interfacial interactions during melt blending.
7. The DSC and DMA results revealed that the addition of UFPNR-SAN had no effect on the Tg of the PLA/UFPNR-SAN composites. Furthermore, the TGA measurements indicated that the UFPNR-SAN particles had no negative influence on PLA’s thermal stability.
8. Overall, these findings highlight the potential of the PLA/UFPNR-SAN composite for applications needing both strength and ductility, especially when bio-based and sustainable materials are a priority.

## Figures and Tables

**Table 1:** Contact angle and surface energy of UFPNR-SAN and PLA.

Sample	Contact Angle (deg)			Surface Energy at 25 °C (mN/m)			Surface Energy at 170 °C (mN/m)		
	DI Water	Di-Iodomethane	Glycerol	*σ^P^*	*σ^d^*	Total	*σ^P^*	*σ^d^*	Total
PLA	53.5 ± 1.3	46.7 ± 1.7	62.4 ±2.0	16.9	29.5	46.4	13.7	24.0	37.7
UFPNR-SAN 1:0	81.7 ± 1.4	75.9 ± 2.2	75.9 ± 3.7	8.5	18.5	27.0	5.2	11.3	16.6
UFPNR-SAN 4:1	57.6 ± 3.0	67.1 ± 1.1	71.7 ± 3.8	20.2	19.0	39.3	14.9	14.1	29.1
UFPNR-SAN 1:1	41.1 ± 4.2	71.2 ± 3.1	66.0 ± 4.9	33.1	16.2	49.2	26.4	12.8	39.3
UFPNR-SAN 1:4	24.9 ±0.8	70.4 ± 1.7	61.6 ± 3.2	42.1	15.8	57.9	35.1	13.2	48.3
UFPNR-SAN 0:1	19.5 ± 2.7	71.4 ± 4.2	59.5 ± 3.4	45.0	15.4	60.4	38.0	13.0	51.0

**Table 2:** Interfacial interaction parameters for UFPNR-SAN particles with PLA at 170 °C.

Sample	Interfacial Tension (mN/m)	Work of Adhesion (mN/m)	Work of Cohesion (mN/m)	Dispersion Factor
UFPNR-SAN 1:0	4.4	49.9	33.1	1.51
UFPNR-SAN 4:1	1.3	65.4	58.1	1.13
UFPNR-SAN 1:1	3.8	73.2	78.5	0.93
UFPNR-SAN 1:4	6.5	79.4	96.5	0.82
UFPNR-SAN 0:1	7.7	81.0	102.0	0.79

**Table 3:** Flexural properties and impact strength results of PLA/UFPNR-SAN blends.

Sample	ST:AN Ratio ^a^	UFPNR Content (wt%)	Flexural Strength (MPa)	Flexural Modulus (GPa)	Impact Strength (KJ/m^2^)
Neat PLA	-	-	105.8 ± 0.92	3.45 ± 0.08	3.56 ± 0.38
PSAN 1:0 (5%) ^b^	1:0	5	90.8 ± 4.65	3.26 ± 0.07	6.5 ± 0.56
PSAN 4:1 (5%)	4:1	5	90.7 ± 0.8	3.32 ± 0.02	8.27 ± 0.73
PSAN 4:1 (10%)	4:1	10	74.6 ± 3.26	2.83 ± 0.07	8.36 ± 0.57
PSAN 4:1 (15%)	4:1	15	63.0 ± 1.86	2.49 ± 0.07	9.34 ± 0.58
PSAN 4:1 (25%)	4:1	25	44.6 ± 2.26	1.81 ± 0.06	12.28 ± 0.65
PSAN 1:1 (5%)	1:1	5	90.2 ± 2.24	3.35 ± 0.08	6.86 ± 0.46
PSAN 1:4 (5%)	1:4	5	89.0 ± 2.25	3.24 ± 0.06	6.09 ± 0.45
PSAN 1:4 (10%)	1:4	10	73.7 ± 1.42	2.92 ± 0.08	7.15 ± 0.75
PSAN 1:4 (15%)	1:4	15	60.8 ± 1.01	2.43 ± 0.07	6.02 ± 0.63
PSAN 1:4 (25%)	1:4	25	37.4 ± 1.86	1.67 ± 0.10	5.92 ± 0.68
PSAN 0:1 (5%)	0:1	5	90.4 ± 3.47	3.15 ± 0.18	5.95 ± 0.37

^a^ Styrene and acrylonitrile ratio used for grafting onto natural rubber. ^b^ PSAN stands for PLA/UFPNR-SAN composite.

**Table 4:** Thermal properties of PLA and PLA/UFPNR-SAN blends determined from DSC first heating scan.

Sample	ST:AN Ratio ^a^	UFPNR (wt%)	T_g_ (°C)	T_m_ (°C)	Δ*H_c_* (J/gr)	Δ*H_m_* (J/gr)	X_c_ (%) ^c^
Neat PLA	-	-	57	151	21.90	21.83	0.0
PSAN 1:0 (5%) ^b^	1:0	5	58	151	19.66	19.74	0.1
PSAN 4:1 (5%)	4:1	5	56	151	22.11	22.35	0.3
PSAN 4:1 (10%)	4:1	10	58	152	19.40	20.10	0.8
PSAN 4:1 (15%)	4:1	15	57	153	17.59	17.84	0.3
PSAN 4:1 (25%)	4:1	25	57	152	17.44	17.79	0.5
PSAN 1:1 (5%)	1:1	5	56	150	21.87	21.90	0.0
PSAN 1:4 (5%)	1:4	5	57	151	20.70	20.86	0.2
PSAN 0:1 (5%)	0:1	5	57	151	18.52	18.60	0.1

^a^ Styrene and acrylonitrile ratio used for grafting onto natural rubber. ^b^ PSAN stands for PLA/UFPNR-SAN composite. ^c^ Degree of crystallinity.

## Data Availability

Data are contained within the article.

## Supplementary Materials

The following supporting information can be downloaded at: https://www.mdpi.com/article/10.3390/polym16162254/s1, Table S1: Density and void content measurements of PLA/UFPNR-SAN composites with different weight contents.
