# Supplementary material for: Toughening Polylactic Acid with Ultrafine Fully Vulcanized Powdered Natural Rubber Graft-Copolymerized with Poly(styrene-co-acrylonitrile): Tailoring the Styrene–Acrylonitrile Ratio for Enhanced Interfacial Interactions
Source: Polymers (Basel). 2024 Aug 8;16(16):2254. doi: 10.3390/polym16162254 (PMC11359756; doi:10.3390/polym16162254)
Supplement: Supplementary file 1 [file polymers-16-02254-s001.zip › polymers-3125933-supplementary.pdf]

Supplementary Information

Toughening Polylactic Acid with Ultrafine Fully Vulcanized Powdered Natural Rubber Graft-Copolymerized with Poly(styrene-co-acrylonitrile): Tailoring the Styrene–Acrylonitrile Ratio for Enhanced Interfacial Interactions

Reza Gholami[1], Ibrahim Lawan[1], Sahar Ebrahimi[1], Achiraya Pattulee[1], Cheol-Hee Ahn[2], Sarawut Rimdusit[1*],

[1] Center of Excellence in Polymeric Materials for Medical Practice Devices, Department of Chemical Engineering, Faculty of Engineering, Chulalongkorn University, Bangkok 10330, Thailand

[2] Department of Materials Science and Engineering, Seoul National University, Seoul 08826, Korea

* Corresponding Author E-mail address: sarawut.r@chula.ac.th

Void content measurements

The potential formation of voids (porosity) during the compression molding of PLA/UFPNR-SAN specimens could influence the results of flexural and impact testing. To ensure the obtained results were not affected by void content or porosity, void content measurements were conducted according to the ASTM D2734 standard method. This method involves calculating void content by comparing the theoretical and measured densities of the composite.

The void content (V) of a polymer composite can be determined using the following equation:

$$V\,(\%) = 100 - \left(\frac{R}{D} + \frac{r}{d}\right) \qquad \text{(Eq. S1)}$$

Where:

- V is the void content (%)
- R and D are the weight content and density of the matrix, respectively
- r and d are the weight content and density of the filler, respectively

The densities of PLA, UFPNR-SAN, and each PLA/UFPNR-SAN composite were measured using a Mettler Toledo Density Kit (MS-DNY-43). This instrument operates by determining the weight of solid specimens in air and a liquid with known density, calculating the solid density based on buoyancy principle. DI water was used for measuring the weight of PLA and

PLA/UFPNR-SAN specimens in the liquid, while ethanol was used for UFPNR-SAN. Five density measurements were performed for each composition, and the average value was reported.

Table S1 summarizes the density measurement results and calculated void content. A slight increase in void content was observed with increasing UFPNR-SAN weight content. However, the maximum value of 1.4% is acceptable for a compression molding process and can be deemed negligible.

Table S1. Density and void content measurements of PLA/UFPNR-SAN composites with different weight contents

Sample	UFPNR-SAN wt%	Measured density (gr/cm^3)	Void content (%)
PLA[a]	-	1.250 ± 0.002	-
UFPNR-SAN[b]	-	0.962 ± 0.005	-
Neat PLA	0	1.248 ± 0.003	0.2
PLA/UFPNR-SAN 5%	5	1.230 ± 0.004	0.2
PLA/UFPNR-SAN 10%	10	1.206 ± 0.002	0.7
PLA/UFPNR-SAN 15%	15	1.183 ± 0.002	1.1
PLA/UFPNR-SAN 25%	25	1.146 ± 0.003	1.4

[a] PLA pellet was used for density measurements

[b] Void free films were prepared by drying fully-vulcanized natural rubber latex in a vacuum oven at 50 °C for 24 hr.
